# Molecules Editorial: Greetings from the Editor-in-Chief

**DOI:** 10.3390/molecules28207171

**Published:** 2023-10-19

**Authors:** Thomas J. Schmidt

**Affiliations:** University of Münster, Institute of Pharmaceutical Biology and Phytochemistry, PharmaCampus, Corrensstrasse 48, D-48149 Münster, Germany; eicmol@uni-muenster.de

Dear readers, authors, reviewers, editors, coworkers and staff of *Molecules*,

it is my immense pleasure to greet you as the journal’s new Editor-in-Chief. In this Editorial, I would like to take the opportunity to introduce myself and share with you some personal thoughts on the journal’s past, present and future.

Born as the son of a chemical technician and a pharmaceutical assistant, it was clear from my youth that I would pursue studying a field related to chemistry. Moreover, being fascinated with biology and other natural sciences, I studied Pharmacy from 1984 to 1989 and then obtained my doctorate (*Doctor rerum naturalium*) in pharmaceutical biology in 1994 at the University of Düsseldorf, Germany. After a brief postdoctoral experience at the Department of Chemistry at LSU (Baton Rouge, LA, USA), I continued the academic track at the University of Düsseldorf and obtained my habilitation and *venia legendi* (license to teach) in 1999. I remained as an Assistant Professor at the same institution from where I then moved on to the University of Münster (Germany) in 2005. Here, I became a University Professor of pharmaceutical biology and phytochemistry, which I have been ever since. In all my scientific career, my work has been dedicated to the structure and biological activity of secondary metabolites of plants. Over more than a decade now, most of this work aimed at finding and characterizing natural products with the potential as leads or drugs against neglected tropical infectious diseases. Among many other activities in research and academia, I became engaged in working with *Molecules* around 2010 and have been the Section Editor-in-Chief (sEiC) for *Natural Products Chemistry* since May of 2016. 

When I was recently asked to take on the position of Editor-in-Chief (EiC) for the whole journal, my first thought—aside from surprise and delight—was “Will this be manageable?” I suppose most scientists working in any field of chemistry would consider it a challenge to be the Chief Editor of an “all chemistry” journal of such a dizzying size as *Molecules*, with almost 9000 papers (articles + reviews) published in 2022. However, having already served the journal for more than seven years as the sEiC of its largest section (with, after all, almost 1800 published papers last year), this concern was relatively easy to soothe. I was already aware of the mechanics with which this “big ship” operated, allowing it to sail smoothly, which was thanks to its well-organized and extremely efficient crew: the editorial staff and system of expert editors. Hence, although the chief editorship role of the whole journal remains to appear as a looming challenge, I look forward to the close collaboration with all the excellent editors of our many different sections. Please be assured that I will rely heavily on your expertise and thank you in advance for your support. I consider it a great honor to be chosen for this position and would herewith like to cordially thank the journal’s administrative management for the confidence of inviting me into this position. I do look forward to a great cooperation with all of the journal’s parts and contributors, especially to the continued smooth and effective collaboration with the editorial staff and journal administration. I am sure that it will be as pleasant and rewarding as it was over the years in my previous position.

When I first became aware of *Molecules* in 2009, the notion of open access publishing was still new to me, but I immediately saw the importance of making scientific results available to every single individual (with internet access) worldwide. Not only did it appear absolutely logical to make research funded by tax money directly accessible to any interested person who paid these taxes, but it also became clear to me that the research results, particularly in the field of neglected tropical diseases—wherein I had recently begun to work—would not be disseminated properly if they were published in journals with availability restricted to the few percent with access to the university libraries of wealthy nations, which are able to afford expensive subscription contracts with large publishing companies. Therefore, I decided to publish my next paper in *Molecules*. Due to the very pleasant experience (the article processing, already at the time—thanks to the excellent editorial office staff—was swift and uncomplicated; and -coincidentally- my first paper later won the *Molecules* “Best Paper Award” (2013)), many more papers followed. One of the main motivations to become increasingly involved in *Molecules* was, and is, my conviction that full open access publishing is the best way of disseminating science. This is why, having been a Reviewer for a while, I also agreed to become part of the journal’s Scientific Editorial Board, and then, in 2016, the sEiC of the *Natural Products Chemistry* section. I can only say today that this has thus far been a very exciting, pleasant, instructive and also rewarding and satisfying journey.

Over the years, I have witnessed a very steady increase in the size of the journal, paralleled also by an increase in the bibliometrics. It was astounding to observe, from period to period, the growth of the journal impact factor (JIF), beginning from a point below 2 (1.74) when I first published in *Molecules* in 2009, up to almost 5 in 2021 (4.6 at present). At the same time, *Molecules* has quite rapidly grown in size, best illustrated by its increasing number of publications, which rose from 362 in 2009 to 8986 in 2022.

The keyword “growth”, in our era of a fast-growing global population on a limited planetary surface, in view of dwindling resources and man-made climate change, along with the paradigm of perpetual (infinite?) growth as the “elixir of life” of the global economy heavily under debate, should be worth some contemplation. Of course, the fast increase in size of *Molecules* in terms of published articles was only possible by steadily increasing the number of scientists contributing as editors and reviewers, and this has evidently worked well for many years. But the number of scientists willing and suitable to serve a journal, of course, is not infinite. Thus, in a similar way as in nature and economy, it may be foreseen that it could become problematic to maintain this kind of journal growth in the long run, because this is likely to affect the scientific quality of the contributions. Along the same lines, there is criticism regarding the speed of publishing, the large number of Special Issues and the financing model of open access journals, whereby authors pay for their publications, which, in the eyes of critics, would make it easier to have substandard work published. These as well as related points of concern must be taken very seriously. Considering that it is the most important task of an Editor to help maintain and improve the scientific quality, I promise that I will continue to conduct my best to counteract any tendencies fostering such concerns.

It is clear that a journal such as *Molecules* (rather similar to the global economy as a whole) must continue “growing” in order to avoid stagnation and dwindling. But this must be healthy growth in order to allow flourishing. The pharmaceutical biologist and phytochemist within myself tends to draw analogies with the plant kingdom: a plant—imagine a big tree—in all its life never stops growing even though it may, from a certain age, cease to grow taller. Healthy growth is characterized by steady change: Old leaves and twigs are lost, but new ones continue to form; older twigs become branches and boughs; and the stem—once a thin stalk—grows into a stronger and wider trunk. This type of growth leads to steady improvement and consolidation. In journal terms, this can be translated to a growth of impact/citations by increasing scientific quality rather than mere growth in the number of articles. The tree, entirely made up of beautiful *Molecules*, grows stronger and more endurable: It does not merely grow, it thrives.

Seeing *Molecules* thrive in this way is my vision for our journal’s bright future.

My sincere regards and good wishes to all.

